# Why Do People Act Like the Proverbial Ostrich? Investigating the Reasons That People Provide for Not Monitoring Their Goal Progress

**DOI:** 10.3389/fpsyg.2017.00152

**Published:** 2017-02-08

**Authors:** Betty P. I. Chang, Thomas L. Webb, Yael Benn

**Affiliations:** ^1^Centre for Research in Cognition and Neurosciences, Centre for Social and Cultural Psychology, Université Libre de BruxellesBruxelles, Belgium; ^2^Department of Psychology, University of SheffieldSheffield, UK; ^3^Department of Psychology, Manchester Metropolitan UniversityManchester, UK

**Keywords:** the ostrich problem, goals, progress monitoring, weight loss, self-weighing

## Abstract

Two studies examined peoples' reasons for not monitoring their progress toward their personal goals—a phenomenon that has been termed “the ostrich problem” (Webb et al., 2013). Study 1 used factor analysis to organize the reasons that people gave for not monitoring their goal progress, resulting in 10 factors. The most strongly endorsed reasons were: (a) that information on goal progress would demand a change in beliefs, or (b) undesired action; (c) that progress was poor, and (d) that thinking about and/or working on the goal was associated with negative emotions. Study 2 adopted a prospective design and investigated whether the reasons identified in Study 1 predicted: (a) the likelihood that participants would decline an opportunity to monitor their goal progress, and (b) the frequency with which participants monitored their goal progress. We found evidence that some of the most strongly endorsed reasons from Study 1 also predicted the avoidance of monitoring in Study 2; however, the belief that information about goal progress was likely to be inaccurate and not useful, and perceived control over goal attainment also reliably predicted the avoidance of monitoring in Study 2. Taken together, the findings explain why people do not monitor their goal progress and point to potential avenues for intervention.

## Introduction

People often want to know how they are doing on their personal goals. For example, someone who has the goal of saving money may look at how much money they have in the bank, someone who has the goal of losing weight may step on a set of scales to see how much they weigh, someone who is trying to get promoted at work may ask their boss for feedback on their work, and someone in a romantic relationship might ask their partner whether they are happy. However, there are also instances where people do *not* seek or pay attention to information about their progress on their personal goals when they could, even if the goal is important to them. For example, sometimes people avoid looking at their bank balance, do not weigh themselves, ask their boss for feedback on their work, or their partner whether they are happy in the relationship. People may not monitor their progress because they are busy or because so doing is not possible, but in some cases they may actually be motivated to avoid or ignore information about their goal progress—a phenomenon that has been referred to as “the ostrich problem” (Webb et al., 2013). Given that monitoring goal progress can promote goal pursuit (for a review, see Harkin et al., 2016), it is important to understand the factors that influence whether or not people monitor their goal progress.

Self-regulation often involves resolving a conflict between competing desires (Emmons et al., 1993). For example, monitoring progress toward the goal of losing weight may require that the person resolves the conflict between wanting to achieve this, and wanting to eat delicious, high calorie foods that might undermine this goal. Therefore, effectively promoting the monitoring of goal progress (e.g., prompting people to weigh themselves regularly) requires identifying both the reasons why people may engage in the behavior (e.g., because it would help them to lose weight) and the reasons why people may not perform it (e.g., because it would make them feel guilty about eating high calorie foods). Because the reasons that people likely have for monitoring or not monitoring their goal progress do not necessarily sit at two ends of the same spectrum (e.g., a strong desire to lose weight does not necessarily entail reduced guilt in response to eating high calorie foods), understanding the former is no substitute for understanding the latter, and both can influence behavior independently of one another. In cases where the reasons for not monitoring exert a stronger effect than the reasons for monitoring (e.g., when the desire to protect the self from negative feedback is stronger than the desire to obtain accurate feedback), designing interventions to promote monitoring also requires an understanding of the reasons why people do not monitor their goal progress. Unfortunately, however, the majority of research to date has focused on why people seek information on their progress (e.g., because it helps them to recognize when additional effort or self-control is needed, Myrseth and Fishbach, 2009; Fishbach et al., 2012), rather than on why they might avoid doing so.

## Why might people avoid monitoring their goal progress?

Several literatures can help to identify reasons why people might not monitor their goal progress. These include studies on why people avoid information more generally (e.g., Sweeny et al., 2010), as well as studies of the effects of feedback in learning and in organizational contexts (e.g., Ilgen et al., 1979; Kluger and DeNisi, 1996; Jordan and Audia, 2012).

One reason that people may have for avoiding or ignoring information about their goal progress is that they do not trust the accuracy of the information that is available (e.g., Ilgen et al., 1979). For example, when feedback is received from others, beliefs about the accuracy of information can be influenced by the perceived expertise of the person providing the feedback (DeBono and Harnish, 1988). The perceived accuracy of the information can also be influenced by the nature of the information. For example, Ilgen et al. found that people are more likely to reject negative than positive feedback, although this relationship is reversed in collectivist cultures (Markus and Kitayama, 1991), and for experts compared to novices (Finkelstein and Fishbach, 2012).

Another factor that may discourage people from monitoring their goal progress is the perceived cost of accessing relevant information (e.g., Ashford and Cummings, 1983; Sweeny et al., 2010). Although information about progress may be useful, obtaining it can be inconvenient, effortful, and/or difficult (Sweeny et al., 2010). For example, it may be effortful to arrange an appointment to see a doctor for an annual check up and find the time to attend. People may also not monitor their goal progress if they believe that the information that they will receive is likely to be of little value (Ashford, 1986). This may be the case if people have difficulty interpreting the information, as when consumers have difficulty understanding nutritional labels and so do not look at them (Cowburn and Stockley, 2005). People may also not see the point of attending to, or accessing, information on their progress if they do not believe that it will help them to achieve their goals. This may be especially likely when people feel that they have relatively little control over the outcome (e.g., as when people are more likely to avoid finding out their risk of a health condition if the condition is untreatable than if it is treatable, Howell and Shepperd, 2012, 2013).

Information about goal progress can suggest that a person needs to change their beliefs. For example, someone who discovers that they have gained weight despite restricting their eating may be forced to conclude that diet is not the only determinant of weight. Given that evidence suggests that people often seek out information that is consistent with their beliefs (an effect that is termed “the confirmation bias”; Nickerson, 1998), they may also ignore or avoid seeking information that could challenge such beliefs (e.g., Adams, 1961). This behavior may be more likely to occur when people are defensive (e.g., they are less confident in their beliefs or are more close-minded; Hart et al., 2009). Information on goal progress may also necessitate action(s) that the person would prefer not to undertake (Sweeny et al., 2010); particularly if the information makes it apparent that the current rate of goal progress is inadequate. For example, someone who discovers that they have gained weight despite restricting their eating may be forced to conclude that they also need to increase the amount of physical activity that they do. In short, people who do not wish to change their beliefs or the way that they pursue their goal may avoid confronting information that could suggest that they should do so.

In addition to the practical costs of obtaining information on goal progress, there may also be affective and motivational costs to obtaining such information. Specifically, evidence suggests that information indicating that progress is worse than expected or desired can lead to unpleasant feelings (Moberly and Watkins, 2010), and so people may be more likely to avoid monitoring their progress if they expect that their progress is relatively poor (cf., Sweeny et al., 2010; Webb et al., 2013). Relatedly, merely thinking about the target goal may make people feel bad, and so people avoid monitoring their goal progress in order to escape from the negative feelings that are associated with the goal (Webb et al., 2013). For example, people with diabetes may avoid monitoring their blood glucose levels (despite the fact that doing so can help them to manage the condition) because self-monitoring requires them to confront the fact that they have diabetes (Candib, 2008).

Finally, it is also possible that people may not monitor their goal progress because doing so may undermine their motivation to achieve their goal. Specifically, evidence suggests that when people are unsure about whether to continue striving for a goal or not (i.e., levels of commitment are relatively low), avoiding information that suggests that progress is poor can guard against further decreases in commitment and prevent disengagement (Finkelstein and Fishbach, 2012). In addition, it is possible that there are occasions in which people might also avoid information that signals good progress because it could lead to complacency and a subsequent decrease in efforts (Amir and Ariely, 2008). Evidence also suggests that people evaluate their progress with respect to a standard that is likely to maintain their motivation (e.g., with respect to their starting point during the early stages of goal pursuit; Bonezzi et al., 2011) and represent their progress in ways that motivate them (e.g., overestimating their progress if they are a long way from achieving their goal; Huang et al., 2012). Taken together then, these findings suggest that people may avoid monitoring their progress for instrumental reasons; namely, to maintain motivation.

## The present research

The following studies examined the reasons why people do not monitor their goal progress. We began by developing a series of statements describing reasons why people might avoid monitoring their goal progress, including: (a) the perceived accuracy and (b) value of the information that is available, (c) the difficulty of obtaining the information, d) the trade-off between the costs and benefits of monitoring progress, (e) the extent to which the person felt that they would be able to understand the information, (f) whether information on goal progress was deemed to be likely to have a negative impact on motivation and/or (g) demand unwanted action or (h) a change in beliefs. The questions also assessed: (i) affect associated with the target goal, (j) affect associated with progress toward the target goal, and (k) perceived control over goal attainment.

Study 1 asked a relatively large sample of participants to think of an occasion when they avoided monitoring their goal progress and to indicate the extent to which each of the statements explained why they did so. Factor analysis was used to organize the reasons before we identified which were most strongly endorsed. Study 2 adopted a prospective design in which participants were offered the opportunity to obtain information about their progress toward a personal goal. However, before participants were offered this opportunity, they rated their beliefs about obtaining information on their goal progress by indicating the extent to which they agreed with each of the statements developed in Study 1. We then examined which beliefs best predicted whether or not participants eschewed the opportunity that they were offered to obtain information on their goal progress.

## Study 1

### Methods

#### Participants and procedure

*N* = 288 participants completed an online questionnaire in response to a request sent to a list of research volunteers at a large university in the UK. Participants were offered the chance to win a £50 Amazon voucher in return for their time. There were relatively equal numbers of males (52%) and females (48%) (1 participant preferred not to specify their gender). Participants were aged between 18 and 66 years (*M* = 27.52, *SD* = 9.80).

In Studies 1 and 2 participants were informed that they could withdraw from the study at any time by closing their browser, and that their responses would remain anonymous (we did not record any personal information from participants with their data). Participants indicated that they agreed to take part in the study by selecting the “I agree” option on the introductory page of the questionnaire. All studies reported here were carried out in accordance with the British Psychological Society's Code of Human Research Ethics^1^. Ethical approval for these studies was obtained from the Department of Psychology Ethics Committee at the University of Sheffield.

#### Measures

Participants were asked to think of a situation in which they were at least somewhat uncertain about their progress toward a particular goal and where they did not pay much attention to information on their progress (e.g., they ignored signs that things were going wrong) or did not seek information on their progress when they could have (e.g., they avoided asking for feedback). They were then asked to briefly describe: (a) the goal that this situation applied to and (b) how they did not pay much attention to information about their progress or did not seek information about their progress when they could have.

Participants were then asked to think about the reasons why they did not monitor their progress toward the stated goal and to rate the extent to which 74 statements reflected these reasons on a 7-point scale anchored by “strongly disagree” and “strongly agree”. Table 1 provides the full list of statements.

**Table 1:** **Principal axis factoring with direct quartimin rotation (Study 1)**.

**Factor/items**	**Loading**
**F1: THINKING ABOUT AND/OR WORKING ON THE GOAL WAS ASSOCIATED WITH NEGATIVE EMOTIONS**	
Thinking about my goal made me feel good (r)	0.84
Thinking about how I might have been doing on this goal made me feel good (r)	0.83
I liked thinking about my goal (r)	0.80
I liked thinking about the issues that my goal was related to (r)	0.77
Thinking about how I might have been doing on this goal was pleasant (r)	0.70
Thinking about how I might have been doing on this goal made me feel calm (r)	0.72
I preferred not to think about my goal	0.68
Thinking about how I might have been doing on this goal made me feel comfortable (r)	0.65
Thinking about how I might have been doing on this goal was unpleasant	0.64^a^
Working on my goal made me feel good (r)	0.63
Thinking about how I might have been doing on this goal made me feel bad	0.62
Thinking about my progress made me feel bad	0.60
Thinking about how I might have been doing on this goal made me feel uncomfortable	0.59^b^
Thinking about how I might have been doing on this goal made me feel anxious	0.47^c^
I did not like thinking about the issues that my goal was related to	0.43
Working on my goal made me feel bad	0.38
% variance	18.09
α	0.94
**F2: INFORMATION ON PROGRESS WAS BELIEVED TO BE INACCURATE**	
I did not trust the information about my progress that was available	0.82
I thought that the information about my goal progress was unreliable	0.81
I felt that the information about my goal progress was likely to be inaccurate	0.81
I thought that the information about my goal progress was not correct	0.76
I thought that the information about my goal progress that was available did not accurately reflect how I was doing	0.67
I felt that the information about my goal progress was too subjective (e.g., I felt that it may have been influenced by personal feelings)	0.56
It was difficult for me to interpret information about my progress	0.50^d^
% variance	11.42
α	0.88
**F3: DISCOVERING THAT PROGRESS IS POOR WOULD HAVE REDUCED MOTIVATION AND EFFORT**	
If I had found out that I was doing poorly, then I would have put less effort into trying to achieve the goal	0.87
If I had found out that I was doing poorly, this would have made me put more effort into the goal (r)	0.87
If I had found out that I was doing poorly, then I would have worked harder on the goal (r)	0.86
If I had found out that I was doing poorly, this would have increased my motivation for the goal (r)	0.85
If I had found out that I was doing poorly, then I would have been more motivated to achieve the goal	0.81
If I had found out that I was doing poorly, this would have prevented me from working on the goal	0.78
If I had found out that I was doing poorly, then I would have been less motivated to achieve the goal	0.76
If I had found out that I was doing poorly, then I would have thought about giving up on this goal	0.72
% variance	8.06
α	0.92
**F4: INFORMATION ON PROGRESS WOULD DEMAND UNDESIRED ACTION**	
Pursuing my goal might have required me to do things that I would rather not do	0.85
Knowing about my progress might have obligated me to do something that I would have preferred not to do	0.84
Finding out about my goal progress might have required me to do something that I didn't want to do	0.81
Information about my progress might have required me to change my behavior in a way that I didn't want to	0.79
I wasn't willing to change the way that I worked on my goal	0.77
If I found out how I was doing, I might have had to change my behavior in a way that I didn't want to	0.69
Changing how I pursued my goal was too difficult	0.59
I didn't want to know if there was a different way that I should be pursuing my goal	0.51
I didn't want to change the way that I worked on my goal	0.52
% variance	6.37
α	0.88
**F5: DISCOVERING THAT PROGRESS IS GOOD WOULD HAVE REDUCED MOTIVATION AND EFFORT**	
If I had found out that I was doing well, this would have made me put more effort into the goal (r)	0.81
If I had found out that I was doing well, this would have increased my motivation for the goal (r)	0.79
If I had found out that I was doing well, then this would have prevented me from working on the goal	0.77
If I had found out that I was doing well, then I would have been more motivated to achieve the goal (r)	0.76
If I had found out that I was doing well, then I would have been less motivated to achieve the goal	0.74
If I had found out that I was doing well, then I would have worked harder on the goal (r)	0.73
If I had found out that I was doing well, then I would have put less effort into trying to achieve the goal	0.70
If I had found out that I was doing well, then I would have thought about giving up on the goal	0.48^e^
% variance	4.84
α	0.87
**F6: INFORMATION ON PROGRESS WAS NOT PERCEIVED TO BE USEFUL**	
I did not see the point of knowing about my goal progress	0.78
I felt that it was unnecessary to know about my goal progress	0.78
I did not think that it was worth seeking information about how I was doing	0.76
Seeking information about how I was doing was not worthwhile	0.72
I did not need to know about my goal progress	0.69
I did not think that I would find information on my goal progress useful in any way	0.69
I felt that what I could gain by knowing about my progress was not worth the cost of obtaining this information	0.52
The cost of obtaining information about my progress was greater than the benefit of knowing the information	0.40^f^
% variance	3.98
α	0.84
**F7: INFORMATION ON PROGRESS WOULD DEMAND A CHANGE IN BELIEFS**	
Knowing about my goal progress would have changed my beliefs about myself and/or my situation	0.73
Knowing about my goal progress would have changed my opinion of myself and/or my situation	0.72
Knowing about my goal progress would have changed the way that I thought about myself and/or my situation	0.70
Knowing about my goal progress would have changed my understanding of myself and/or my situation	0.66
% variance	3.50
α	0.85
**F8: INFORMATION ON PROGRESS WAS DIFFICULT TO OBTAIN AND/OR UNDERSTAND**	
Information about how I was doing was hard to come by	0.84
Finding information about how I was doing was difficult	0.82
Information about how I was doing was not freely available	0.76
It was hard for me to find information about my goal progress	0.74
It was too much effort to obtain information about my progress	0.71
There was no clear way for me to know how I was doing	0.49^g^
It was not easy for me to understand information about my progress	0.42
% variance	2.75
α	0.88
**F9: PROGRESS WAS POOR**	
My progress was worse than I had hoped	0.77
My progress was at least as good as I had hoped (r)	0.76
I was making good progress (r)	0.68
My progress was slower than I would have liked it to be	0.53
% variance	2.37
α	0.80
**F10: LACK OF CONTROL OVER GOAL ATTAINMENT**	
I felt that whether I achieved the goal or not depended on factors outside of my control	0.84
I felt that I had little control over whether I achieved the goal or not	0.84
I completely influenced whether I achieved the goal or not (r)	0.65
% variance	2.17
α	0.74

a *Cross loads on F7 with a loading of 0.30*.

b *Cross loads on F7 with a loading of 0.31*.

c *Cross loads on F7 with a loading of 0.41*.

d *Cross loads on F8 with a loading of 0.36*.

e *Cross loads on F6 with a loading of 0.31*.

f *Cross loads on F8 with a loading of 0.33*.

g *Cross loads on F2 with a loading of 0.32*.

### Results

The goals that participants reported avoiding monitoring related to physical activities or health (25%, e.g., a participant described trying to lose weight, but reported that they avoided weighing themselves), money and finances (15%, e.g., a participant reported trying to pay off their credit card debt, but that they left their credit card statements unopened), work or study (52%, e.g., a participant reported having the goal of doing well at university, but did not read feedback on their work), relationships and social goals (1%, e.g., a participant reported wanting to have a good romantic relationship, but did not ask their partner about how they felt about the relationship), or other goals (7%, e.g., a participant reported having the goal to manage their time better, but did not keep a diary). The descriptions of 58 participants (20%) did not reflect situations where they did not pay attention to, or seek information on their progress (e.g., these participants typically described how they did not take action to strive for their goal) and so these participants were excluded from subsequent analyses.

Factor analysis was used to investigate the conceptual structure of the statements reflecting why participants did not monitor their goal progress. The Kaiser-Meyer-Olkin measure of sampling adequacy (0.84), and Bartlett's test of sphericity (11608.49, *df* = 2701, *p* < 0.001) indicated that the correlation matrix was appropriate for factor analysis (Dziuban and Shirkey, 1974; Kaiser and Rice, 1974). Principal factor extraction with direct quartimin rotation was therefore performed through SPSS on 74 items. Initially 15 factors were extracted (based on Kaiser's, 1958, criterion) that explained 71.49% of the variance in participants' responses. However, because this factor structure resulted in three factors that comprised of only two statements each and one factor that comprised only of cross-loadings, a 10 factor structure that was empirically coherent and made conceptual sense was extracted that explained 63.55% of the variance in responses. Table 1 shows the loadings of variables on the factors, the amount of variance explained by each factor, and the alpha coefficient representing the internal reliability of each factor. Variables are ordered by the size of their loading on their corresponding factor in order to facilitate interpretation.

Factor 1 had high loadings from statements reflecting the idea that participants did not pay attention to, or seek information about, their goal progress because thinking about and/or working toward the goal was associated with negative emotions. Factor 1 was therefore labeled “*Thinking about and/or working on the goal was associated with negative emotions*.” The statements that loaded on Factor 2 reflected the belief that the information that was available on goal progress was likely to be inaccurate and were therefore labeled “*Information on progress was believed to be inaccurate*.” The statements that loaded on Factor 3 reflected the idea that participants did not monitor their goal progress because they felt that if they discovered that their progress was poor then it would have undermined their motivation to achieve the goal or the amount of effort that they were willing to exert in order to achieve the goal. Factor 3 was therefore labeled “*Discovering that progress is poor would have reduced motivation and effort*.” Factor 5 reflected a similar idea, but with respect to the discovery that progress was good and so was labeled “*Discovering that progress is good would have reduced motivation and effort*.”

Factor 4 had high loadings from statements reflecting the idea that “*Information on progress would demand undesired action*,” and so was labeled accordingly. The statements that loaded on Factor 6 reflected the idea that participants did not monitor their goal progress because the information likely to be obtained was not deemed to be useful, and so Factor 6 was labeled “*Information on progress was not perceived to be useful*.” Statements suggesting that knowing about goal progress would have led to a change in beliefs loaded on Factor 7, which was therefore labeled “*Information on progress would demand a change in beliefs*.” The statements that loaded on Factor 8 reflected the idea that “*Information on progress was difficult to obtain or understand*” and so Factor 8 was labeled accordingly. The statements that loaded on Factor 9 epitomized the idea that people do not monitor their progress because they expect that doing so would reveal that their progress was relatively poor. Factor 9 was therefore labeled “*Progress was poor*.” Factor 10 was labeled “*Lack of control over goal attainment*” because the three statements loading on this factor reflected (a lack of) perceived behavioral control over goal attainment.

There were relatively few cross loadings (just six items loaded >0.30 on more than one factor) and all of the factors proved internally reliable (median α = 0.85, range 0.74–0.94). Scale scores were computed by averaging items loading on the relevant factor^2^.

Table 2 shows the correlations between the factors. For the most part, the correlations were relatively small (mean of absolute values of *r* = 0.17, minimum *r* = −0.29, maximum *r* = 0.55) supporting the discriminant validity of the factors. Only one correlation exceeded Cohen's (1992) criteria for a “large” correlation (*r* > 0.50); this was between Factor 2: *Information on goal progress was believed to be inaccurate* and Factor 8: *Information on goal progress was difficult to obtain and/or understand* (*r* = 0.55). This correlation is intelligible as each of these factors represented the challenges of obtaining and interpreting information on goal progress. However, the correlation is not so high as to suggest that these factors measure the same construct (i.e., that the factors are multicollinear).

**Table 2:** **Correlations between factors (Study 1)**.

	**F1**	**F2**	**F3**	**F4**	**F5**	**F6**	**F7**	**F8**	**F9**
F2	0.03								
F3	**0.34**	−0.01							
F4	**0.41**	0.04	**0.32**						
F5	0.12	−0.03	**0.22**	0.04					
F6	−0.09	0.00	**0.20**	0.08	**0.24**				
F7	**0.37**	0.01	**0.24**	**0.43**	0.03	**0.34**			
F8	−0.10	**0.55**	−**0.16**	0.01	−0.03	**0.26**	−0.06		
F9	**0.48**	−0.06	**0.20**	**0.15**	0.01	−**0.29**	**0.16**	−**0.14**	
F10	**0.27**	**0.38**	0.06	0.06	0.05	**0.19**	**0.13**	**0.17**	0.09

*Correlations in bold are significant at p < 0.05*.

## Which reasons for not monitoring goal progress did participants most strongly endorse?

In order to investigate which reasons for not monitoring goal progress were most strongly endorsed by participants, we computed descriptive statistics (see Table 3) and then ran a repeated measures ANOVA with factor as the within-participants variable. As expected, there were significant differences in the extent to which participants endorsed different reasons for not monitoring their goal progress, *F*_(9, 212)_ = 49.99, *p* < 0.001, eta^2^ = 0.68. Pairwise comparisons (with Bonferroni adjustment) were used to identify which reasons were differentially endorsed (indicated by subscripts in Table 3). The most strongly endorsed reasons for not monitoring goal progress were that information on goal progress would demand: (a) a change in beliefs (Factor 7: *M* = 4.76, *SD* = 1.30) or (b) undesired action (Factor 4: *M* = 4.73, *SD* = 1.13). The next most strongly endorsed reasons for not monitoring were: (c) that progress was likely to be poor (Factor 9: *M* = 4.50, *SD* = 1.21), and (d) that thinking about and/or working on the goal was associated with negative emotions (Factor 1: *M* = 4.41, *SD* = 1.19).

**Table 3:** **Descriptive statistics (*M* and *SD*) for factor scores by goal domain and overall (Study 1)**.

**Factor**	**Physical/health**	**Finance**	**Work/study**	**Social/relationships**	**Other**	**Overall**
	**(*N* = 60)**	**(*N* = 37)**	**(*N* = 107)**	**(*N* = 2)**	**(*N* = 15)**	**(*N* = 221)**
F7: Information on progress would demand a change in beliefs	4.85 (1.32)	4.51 (1.35)	4.82 (1.26)	5.25 (0.35)	4.50 (1.47)	4.76 (1.30)_a_
F4: Information on progress would demand undesired action	4.79 (1.13)	4.66 (1.00)	4.77 (1.12)	4.94 (0.86)	4.42 (1.37)	4.73 (1.13)_a_
F9: Progress was poor	4.82 (1.02)	4.73 (1.18)	4.29 (1.25)	4.38 (1.94)	4.35 (1.29)	4.50 (1.21)_ab_
F1: Thinking about and/or working on the goal was associated with negative emotions	4.53 (1.00)	4.44 (1.21)	4.42 (1.25)	3.66 (1.10)	3.87 (1.41)	4.41 (1.19)_b_
F3: Discovering that progress is poor would have reduced motivation and effort	4.47 (1.32)	3.80 (1.33)	3.63 (1.33)	2.56 (0.97)	3.95 (1.29)	3.87 (1.37)
F10: Lack of control over goal attainment	3.03 (1.20)	3.60 (1.21)	3.60 (1.49)	5.50 (1.65)	3.40 (1.35)	3.46 (1.39)_c_
F6: Information on progress was not perceived to be useful	3.23 (0.97)	3.29 (1.13)	3.56 (1.27)	3.39 (1.02)	3.23 (0.88)	3.33 (1.07)_cd_
F2: Information on progress was believed to be inaccurate	2.89 (1.18)	2.70 (1.11)	3.42 (1.14)	4.36 (0.91)	3.53 (1.45)	3.19 (1.30)_cd_
F5: Discovering that progress is good would have reduced motivation and effort	3.10 (1.02)	3.29 (1.13)	3.42 (1.32)	1.88 (0.18)	2.93 (1.17)	3.18 (1.10)_cd_
F8: Information on progress was difficult to obtain/understand	2.56 (1.15)	2.66 (1.10)	3.23 (1.11)	4.86 (1.62)	3.21 (1.39)	3.16 (1.31)_d_

*Factors are ordered by highest overall mean first. Pairwise comparisons with Bonferroni adjustment were used to compare overall means. Means that share subscripts do not differ significantly*.

### Discussion

Study 1 found that the reason that participants most strongly endorsed for not monitoring their goal progress was that doing so might demand a change in beliefs and/or warrant undesired action(s). Participants also reported that feeling negatively about their goal and/or their progress toward that goal discouraged them from monitoring their progress. Reasons that were more cognitive (e.g., that information on goal progress would not be useful or accurate) were less strongly endorsed, as were structural reasons for not monitoring (e.g., that information on goal progress was difficult to obtain and/or understand). These findings provide an initial suggestion that the reasons people have for not monitoring may be more emotional, rather than practical in nature.

The findings of Study 1, while informative, are based on participants' retrospective reports of why they did not monitor their progress. It may therefore be that participants more strongly endorsed emotional reasons for not monitoring their progress not because such reasons actually motivated their behavior, but because emotional reasons tend to command more attention (e.g., Öhman et al., 1995; Vuilleumier et al., 2001) and be more memorable (e.g., Sharot and Phelps, 2004) than non-emotional reasons. As such, participants may have more readily attributed their lack of monitoring to emotional causes rather than to more mundane reasons for avoiding monitoring. Indeed, Nisbett and Wilson (1977) argue that people's explanations for their behavior rely more on their beliefs about plausible causes rather than on careful introspection. To address this potential alternative interpretation of our findings, Study 2 adopted a prospective design and investigated whether the reasons identified in Study 1 predicted the likelihood that participants would decline an opportunity to monitor their goal progress.

## Study 2

Study 2 offered participants who were currently trying to lose weight the opportunity to monitor their progress toward this goal. This was achieved by creating an online “progress calculator” that allowed participants to input information about their weight loss goal and how they pursued it, after which they could ostensibly receive information on whether their current approach would allow them to attain their goal or not. In addition, we asked participants how frequently they weighed themselves as a measure of the extent to which they monitored their progress toward their goal of losing weight in their everyday lives.

To investigate the predictive value of the reasons that participants may have for not monitoring their progress, we first asked participants to indicate how strongly they felt that each of the factors identified in Study 1 pertained to their goal of losing weight and/or monitoring their progress toward this goal, without reference to whether this factor was responsible for their (lack of) progress monitoring (e.g., we asked participants whether they associated trying to lose weight with negative emotions, not whether feeling bad about trying to lose weight explained why they did not monitor their progress toward this goal). We then investigated whether variations in the strength of these factors predicted: (a) whether participants accessed the progress calculator, and (b) the frequency with which they monitored their progress toward the goal of losing weight by self-weighing. For comparison with Study 1, we also examined the extent to which participants endorsed each of the reasons that might explain why they did not monitor their goal progress. Finally, we investigated whether participants who weighed themselves less frequently (and thus might be assumed to avoid monitoring their progress) endorsed these reasons to a greater extent than participants who weighed themselves more frequently.

### Method

#### Participants

An email was sent to a list of volunteers at a large university in the UK, inviting people who were currently trying to lose weight to complete a survey about their thoughts and feelings about so doing. Eight hundred and thirty-two people volunteered for the study and were offered the chance to win one of two £50 Amazon vouchers. Test questions (that instructed participants to select a particular response) were randomly placed in the survey to ensure the integrity of the data. Participants were excluded if they indicated that they were only thinking about losing weight but were not currently working on the goal (*n* = 134), and/or if they answered at least one of these test questions incorrectly (*n* = 64). This left *n* = 634 participants—451 females (73%), 166 males (27%), and 3 (< 1%) who identified their gender as “other.” Fourteen participants (2%) did not report their gender. Data on the age of two participants was excluded because they reported that their age was either 1 or 95. The remaining participants were aged between 18 and 66 years (*M* = 27.04, *SD* = 10.31).

#### Procedure

Participants were first asked to describe their goal for losing weight (e.g., I want to lose 10 kilos in 2 months; I want to fit into size 10 clothing). They also indicated how frequently they weighed themselves using a scale developed by Klos et al. (2012): 1 = Daily; 2 = 3–5 times a week; 3 = 1–2 times a week; 4 = Once a fortnight; 5 = Once a month; 6 = Once every 3 months, and 7 = less than once every 3 months.

Next, participants answered questions designed to assess each of the 10 reasons identified in Study 1 as reasons why people might not monitor their goal progress. Where possible, these questions were based on the 4 statements that had the highest loadings on each factor, but were adapted to refer to how participants currently thought or felt about their weight loss goal (instead of the reasons why participants did not monitor their goals, as in Study 1). For example, the statement “My progress was slower than I would have liked it to be” was rephrased to “My progress toward my weight loss goal is probably slower than I would like it to be.” These statements are presented in Table 4, along with the internal reliability of the resulting measures. Participants indicated the extent to which they agreed with each statement on a scale of 1 = strongly disagree to 7 = strongly agree. The remaining 34 items from Study 1 were omitted in order to reduce the length of the questionnaire.

**Table 4:** **Principal axis factoring with direct Quartimin rotation (Study 2)**.

**Factor/items**	**Loading**
**F1: THINKING ABOUT AND/OR WORKING ON THE GOAL IS ASSOCIATED WITH NEGATIVE EMOTIONS**	
Thinking about trying to lose weight makes me feel good (r)	0.86
I like thinking about trying to lose weight (r)	0.88
Thinking about trying to lose weight makes me feel anxious	0.46
% variance	5.78
α	0.70
Thinking about how much weight I have lost makes me feel good (r)^a^	
**F2: INFORMATION ON PROGRESS IS BELIEVED TO BE INACCURATE**	
I do not trust the information that is available about my progress toward my weight loss goal	0.81
The information that is available about my progress toward my weight loss goal is unreliable	0.77
The information that is available about my progress toward my weight loss goal is likely to be accurate (r)	0.83
The information that is available about my progress toward my weight loss goal is likely to be incorrect	0.81
% variance	11.42
α	0.88
**F3: DISCOVERING THAT PROGRESS IS POOR WOULD REDUCE MOTIVATION AND EFFORT**	
If I discovered that I was not making good progress toward my weight loss goal, then I would put less effort into trying to lose weight	0.75
If I found out that I was not making good progress toward my weight loss goal, then I would put more effort into trying to lose weight (r)	0.81
If I discovered that I was not making good progress toward my weight loss goal, then I would work harder to achieve my weight loss goal (r)	0.79
If I found out that I was not making good progress toward my weight loss goal, then this would increase my motivation to lose weight (r)	0.81
% variance	4.95
α	0.83
**F4: INFORMATION ON PROGRESS WOULD DEMAND UNDESIRED ACTION**	
Trying to lose weight might require me to do things that I would rather not do	0.85
Knowing about my progress toward my weight loss goal might obligate me to do something that	
I would prefer not to do	0.84
Finding out about my progress toward my weight loss goal might require me to do something that	
I don't want to do	0.81
Information about my progress toward my weight loss goal might require me to change my behavior in a way that I don't want to	0.79
% variance	10.53
α	0.82
**F5: DISCOVERING THAT PROGRESS IS GOOD WOULD REDUCE MOTIVATION AND EFFORT**	
If I discovered that I was making good progress toward my weight loss goal, then I would put less effort into trying to lose weight	0.76
If I found out that I was making good progress toward my weight loss goal, then this would increase my motivation to lose weight (r)	0.78
If I discovered that I was making good progress toward my weight loss goal, then this would prevent me from trying to lose weight	0.73
If I found out that I was making good progress toward my weight loss goal, then I would be more motivated to lose weight (r)	0.80
% variance	4.84
α	0.87
**F6: INFORMATION ON PROGRESS IS NOT PERCEIVED TO BE USEFUL**	
I do not see the point of knowing about my progress toward my weight loss goal	0.72
I feel that it is unnecessary to know about my progress toward my weight loss goal	0.66
I do not think that it is worth seeking information about my progress toward my weight loss goal	0.83
Seeking information about my progress toward my weight loss goal is not worthwhile	0.77
% variance	3.55
α	0.78
**F7: INFORMATION ON PROGRESS WOULD DEMAND A CHANGE IN BELIEFS**	
Knowing about my progress toward my weight loss goal would change my beliefs about myself and/or my situation	0.84
Knowing about my progress toward my weight loss goal would change my opinion of myself and/or my situation	0.87
Knowing about my progress toward my weight loss goal would change the way that I think about myself and/or my situation	0.83
Knowing about my progress toward my weight loss goal would change my understanding of myself and/or my situation	0.79
% variance	19.34
α	0.84
**F8: INFORMATION ON PROGRESS IS DIFFICULT TO OBTAIN AND/OR UNDERSTAND**	
Information about my progress toward my weight loss goal is hard to come by	0.83
Finding information about my progress toward my weight loss goal is difficult	0.83
Information about my progress toward my weight loss goal is not freely available	0.87
It is hard for me to find information about my progress toward my weight loss goal	0.84
% variance	3.82
α	0.91
**F9: PROGRESS IS POOR**	
My progress toward my weight loss goal is likely to be poor	0.77
My progress toward my weight loss goal is likely to be at least as good as I had hoped (r)	0.78
I think that I am making good progress toward my weight loss goal (r)	0.81
My progress toward my weight loss goal is probably slower than I would like it to be	0.74
% variance	9.48
α	0.83
**F10: LACK OF CONTROL OVER GOAL ATTAINMENT**	
Whether I achieve my weight loss goal or not depends on factors outside of my control	0.72
I have little control over whether I achieve my weight loss goal or not	0.80
I have a lot of influence over whether I achieve my weight loss goal or not (r)	0.78
I control whether I achieve my weight loss goal or not	0.82
% variance	2.94
α	0.81

a *This variable was removed from the overall analyses because it did not load on any factor*.

Following this, participants provided demographic information. They were then told that they had reached the end of the questionnaire, but that they had the option of using an online tool that could indicate whether they were on track to achieving their weight loss goal. Participants were told that, in order to calculate their progress, they would have to input their desired weight, current weight, the number of minutes of physical activity that they undertook each week, and what food they ate on an average day. Participants were informed that this would take around 10 minutes and that, based on this information, the calculator would assess whether they were on track to achieving their goal. Our primary dependent variable was whether participants accessed the progress calculator^3^.

### Results

Factor analysis was used to examine whether the measures conformed to the anticipated 10 factor structure (see Table 4). The Kaiser-Meyer-Olkin measure of sampling adequacy was 0.84, and Bartlett's test of sphericity was 11600.36, *df* = 780, *p* < 0.001, suggesting that the data was suitable for analysis. Principal factor extraction with direct quartimin rotation was therefore performed through SPSS on the 40 items. Ten factors were extracted (based on Kaiser's, 1958, criterion) that explained 67.46% of the variance in participants' responses. All items loaded exclusively on the factor that they were designed to measure, except for one question (“Thinking about how much weight I have lost makes me feel good”), which did not load on any of the 10 factors and thus was removed from further analyses.

12.6% of the participants reported weighing themselves daily, 13.9% weighed themselves between 3 and 5 times a week, 30.4% weighed themselves once or twice a week, 15.0% weighed themselves once a fortnight, 10.9% weighed themselves once a month, 5.2% weighed themselves once every 3 months, and 12.0% weighed themselves less than once every 3 months.

## Which reasons for not monitoring goal progress did participants most strongly endorse?

Table 5 shows the descriptive statistics for each factor. A repeated measures ANOVA with factor as the within-participants variable indicated that there was a significant difference in the extent to which different beliefs were endorsed, *F*_(7.28, 623)_ = 459.68, *p* < 0.001, partial eta^2^ = 0.42. Pairwise comparisons (with Bonferroni adjustment) indicated that, overall, participants felt most strongly that: (a) information on goal progress would demand a change in beliefs (Factor 7: *M* = 4.91, *SD* = 1.17). The next most strongly endorsed beliefs were that: (b) information would demand undesired action (Factor 4: *M* = 4.34, *SD* = 1.31), (c) progress is poor (Factor 9: *M* = 4.17, *SD* = 1.15), and (d) thinking about and/or working on the goal was associated with negative emotions (Factor 1: *M* = 3.58, *SD* = 1.16). Participants less strongly endorsed the belief that discovering that their progress was poor would reduce their motivation and effort (Factor 3: *M* = 3.21, *SD* = 1.24), that information about their progress was likely to be inaccurate (Factor 2: *M* = 3.01, *SD* = 1.14), that information about their progress was difficult to obtain and/or understand (Factor 8: *M* = 2.99, *SD* = 1.33), that the information was unlikely to be useful (Factor 6: *M* = 2.44, *SD* = 1.01), or that they lacked control over goal attainment (Factor 10: *M* = 2.39, *SD* = 1.06). Participants also did not believe that discovering that they were making good progress would reduce their motivation or effort (*M* = 2.33, *SD* = 0.92).

**Table 5:** **Descriptive statistics (M and SD) for factor scores (Study 2)**.

**Factor**	***M* (*SD*)**
F7: Information on progress would demand a change in beliefs	4.91 (1.17)
F4: Information on progress would demand undesired action	4.34 (1.31)_a_
F9: Progress is poor	4.17 (1.15)_a_
F1: Thinking about and/or working on the goal is associated with negative emotions	3.58 (1.16)_b_
F3: Discovering that progress is poor would reduce motivation and effort	3.21 (1.24)_c_
F2: Information on progress is believed to be inaccurate	3.01 (1.14)_c_
F8: Information on progress is difficult to obtain and/or understand	2.99 (1.33)_c_
F6: Information on progress is not perceived to be useful	2.44 (1.01)_d_
F10: Lack of control over goal attainment	2.39 (1.06)_d_
F5: Discovering that progress is good would reduce motivation and effort	2.33 (0.92)_d_

*Factors are ordered by highest overall mean first. Pairwise comparisons with Bonferroni adjustment were used to compare overall means. Means that share subscripts do not differ significantly*.

## Which beliefs predict whether participants decline the opportunity to monitor their progress?

To examine which of the factors predicted whether participants' declined the opportunity to use the progress calculator, we conducted a logistic regression with avoidance of the progress calculator (1 = yes, 0 = no) as the dependent variable and the 10 factors as the predictors. The results of this analysis are presented in Table 6. Participants were more likely to decline the opportunity to use the progress calculator when they doubted the accuracy of information [B = 0.20, *S.E*. = 0.09 $\chi_{\left(10,\;632\right)}^{2}$ = 4.26, *p* = 0.04, *odds ratio* = 0.82], when they did not think that it would be useful to have information about their goal progress [B = 0.29, *S.E*. = 0.10 $\chi_{\left(10,\;632\right)}^{2}$ = 8.20, *p* = 0.01, *odds ratio* = 0.75], or when they felt that they had more control over their weight loss [B = −0.23, *S.E*. = 0.09 $\chi_{\left(10,\;632\right)}^{2}$ = 6.73, *p* = 0.009, *odds ratio* = 1.26].

**Table 6:** **Predictors (rows) of avoiding monitoring goal progress (Study 2)**.

**Factor**	**Declined to use the progress calculator (B, *S.E*.)**	**Infrequent self-weighing (*B*)**
F1: Goal is associated with negative emotions	0.08 (0.08)	0.14^**^
F2: Information is believed to be inaccurate	0.20^*^ (0.09)	−0.01
F3: Poor progress would reduce motivation/effort	0.05 (0.08)	−0.04
F4: Information would demand undesired action	0.03 (0.07)	−0.11^**^
F5: Good progress would reduce motivation/effort	0.06 (0.10)	−0.07
F6: Information is not perceived to be useful	0.29^**^ (0.10)	0.38^***^
F7: Information would demand a change in beliefs	−0.04 (0.08)	−0.08
F8: Information is difficult to obtain/understand	−0.14 (0.08)	0.08
F9: Progress is poor	0.00 (0.09)	0.06
F10: Lack of control over goal attainment	−0.23^**^ (0.09)	−0.19^***^
Constant	−0.29	3.05
Model χ^2^/*F*	27.27	14.21
Nagelkerke **R**^2^/**R**^2^	0.06	0.18

* *p < 0.05*,

** *p < 0.01*,

*** *p < 0.001*.

## Which beliefs predict the frequency of self-weighing?

We also examined which of the factors predicted whether participants would weigh themselves as a means of assessing their progress toward their goal of losing weight (see Table 6). A multiple linear regression revealed that participants weighed themselves less frequently if they felt negatively about their goal [*B* = −0.21, *t*_(10, 632)_ = 3.29, *p* = 0.001, *C.I*. = 0.09–0.34], but they weighed themselves more frequently when they felt that information about goal progress would demand undesired action [*B* = 0.15, *t*_(10, 632)_ = 2.72, *p* = 0.007, *C.I*. = −0.26 to −0.04]. As with the progress calculator, participants weighed themselves less frequently when they did not feel that information about their goal progress would be useful [*B* = 0.68, *t*_(10, 632)_ = 8.77, *p* < 0.001, *C.I*. = 0.52–0.83], and felt that they had more control over their goal attainment [*B* = −0.33, *t*_(10, 632)_ = −4.59, *p* < 0.001, *C.I*. = −0.46 to −0.19].

### Discussion

Study 2 adopted a prospective design and measured participants' thoughts and feelings about losing weight and monitoring their progress toward this goal before offering them the opportunity to obtain information about their progress. The findings of Study 2 supported those of Study 1 in showing that participants endorsed the idea that information on their progress would likely demand a change in their beliefs and/or actions, that their progress was likely to be poor, and that thinking about and/or working on the goal was associated with negative emotions. The extent to which the goal was associated with negative emotions predicted the frequency with which participants weighed themselves in order to monitor their progress. Surprisingly, the more that participants thought that monitoring their progress would demand undesired action, the more they weighed themselves. This finding suggests that at least some participants may have weighed themselves in an effort to inform goal pursuit (e.g., to identify whether they needed to change their energy intake or expenditure), even when they would prefer not to make changes to these behaviors.

Participants were less likely to weigh themselves or, to take the opportunity to monitor their progress if they felt that information about their progress was unlikely to be useful and if they felt greater control over their goal attainment. Finally, endorsing the belief that information about goal progress was likely to be inaccurate was associated with declining the opportunity to use the progress calculator, but was not associated with the frequency of self-weighing, perhaps because the information obtained from self-weighing is considered to be objectively accurate.

### General discussion

Research suggests that there are a myriad of different reasons why people do not monitor their progress toward their personal goals. In order to identify the conceptual structure of such reasons and which reasons explain why people do not monitor their goal progress, we developed a set of statements that reflect possible reasons. We then asked two large samples of participants to indicate the extent to which each reason explained a recent instance in which they did not monitor their goal progress (Study 1) or reflected their beliefs about monitoring their progress toward their goal of losing weight (Study 2). Study 2 also provided participants with an opportunity to obtain information about their progress and examined which beliefs predicted whether they would decline that opportunity.

Study 1 identified 10 reasons why people did not monitor their goal progress, including that thinking about and/or working on the goal was associated with negative emotions, that information on progress was not perceived to be useful, and that discovering that progress was poor would have reduced motivation and effort. Study 2 revealed that participants who were trying to lose weight avoided monitoring their progress toward this goal (either by not weighing themselves or by declining the opportunity to use an online calculator) when they felt that information on their progress was unlikely to be useful. This finding is consistent with previous research showing that people are more likely to seek information that they believe will help their goal progress (e.g., information that might allow them to improve, rather than assess their weaknesses, Trope et al., 2003). Participants in Study 2 may not have found the methods for monitoring progress that we assessed (namely, the opportunity to use online calculator and self-weighing) to be useful because they did not know how to make use of the information derived from them to inform goal pursuit. This idea is supported by a study by Mintz et al. (2013), which found that only 60% of female college students who weighed themselves reported that they did so because it helped them to manage their weight, suggesting that 40% did not make use of the information that they derived from self-weighing. This implies that interventions designed to promote progress monitoring may need to address the reasons why people may not think that monitoring progress is useful, and help them to make use of the information derived from monitoring.

The findings of Study 2 suggest that people were less likely to monitor their progress when they associated the goal and/or monitoring their progress toward that goal with negative emotions. Avoiding monitoring may serve as a way of disengaging from the goal, which is consistent with the ideas of Control theory (Carver and Scheier, 1982, 1990) that people may abandon goal pursuit when they feel that progress is not going well and is not expected to improve (see also Campion and Lord, 1982; Louro et al., 2007; Wang and Mukhopadhyay, 2012). This may also allow individuals to regulate their emotions, as by ignoring their goal progress they minimize their exposure to the negative emotions associated with the target goal. Moreover, people may also wish to avoid monitoring when they feel negatively about their goal because certain methods of assessing progress, such as self-weighing, can have further negative affective and psychological consequences (e.g., Ogden and Whyman, 1997; Mintz et al., 2013; for a review, see Benn et al., 2016).

Finally, participants also tended to avoid monitoring when they felt in control of their goal attainment. At first glance, this relationship may seem to contradict the findings of Howell and Shepperd (2012, 2013) who found that people were more likely to avoid information about their risk of a disease when they felt that they had no control over that disease (i.e., when the disease was presented as being untreatable). However, participants in Study 2 generally felt that their weight was controllable to some extent, which raises the possibility that they may have monitored their progress in an effort to gain further control. Monitoring progress may provide control (or at least the illusion of control) because it can provide information that helps people to solve problems, make decisions, and direct action and efforts (Myrseth and Fishbach, 2009; Fishbach et al., 2012)—all processes that can be used to gain control over stressful situations (Folkman, 1984). The discussion above therefore suggests that the relationship between level of control and progress monitoring may have an inverted U-shaped function, such that very low and high levels of control are associated with reduced monitoring, but that more average levels of control are associated with greater monitoring.

## Limitations

It is worth acknowledging a few limitations to the present work. First, Study 1 was retrospective to the extent that participants were asked to recall a time when they did not monitor their goal progress, and to explain the reason for their inaction. This method may have led participants to attribute their lack of progress monitoring to aspects of the situation that were most memorable (such as the emotions that they experienced in that situation). To overcome this limitation, Study 2 adopted a prospective design that allowed us to assess which factors predicted a behavioral and a self-report measure of the avoidance of monitoring. Our finding that some of the factors that best predicted why participants avoided monitoring—e.g., that information on progress monitoring was considered to be of little use, that participants felt in control of their goal attainment—were not always those that were most strongly endorsed as explanations for their lack of monitoring by the participants themselves, suggests that peoples' explanations of why they do not monitor their goal progress may not accurately reflect why they actually avoid monitoring their progress. Self-presentation concerns aside, it is possible that some people may not be aware of their actual reasons for avoiding monitoring. In support of this idea, Mintz et al. (2013) found that 7.7% of participants admitted that they did not know why they weigh themselves.

Another possible explanation for why the most strongly endorsed reasons for not monitoring in Study 1 did not predict the lack of monitoring in Study 2 may be that the a posteriori thought processes that were activated in Study 1, when participants were asked to explain why they avoided monitoring, are different to those that were invoked in Study 2, when participants were asked to consider whether they wanted information on their goal-progress in a specific situation. Specifically, the procedure of Study 1 may have led participants to focus on their motivations, while the procedure of Study 2 may have led participants to focus more on the quality of the information that was available on their progress and the current state of their goal pursuit^4^.

The correlational nature of Study 2 is also a limitation, and prevents us from drawing strong conclusions about the extent to which holding particular beliefs is causally related to people not monitoring their goal progress. However, the relationships we found are consistent with experimental research, such as that showing that the perceived usefulness of feedback influences whether or not it is accessed (Trope et al., 2003), and that people may search for information as a way of gaining control over a situation (Miller, 1987). Furthermore, it may be difficult to experimentally manipulate factors related to monitoring progress toward losing weight because beliefs about losing weight and the emotions associated with such a goal are often long-held and thus not easily changed.

## Conclusion

Although people often wish to know how they are doing on their personal goals, there are also instances when people do not pay attention to or seek information about their progress, even if the goal is important to them—a phenomenon that has been termed “the ostrich problem” (Webb et al., 2013). The present research highlights the multiple reasons why people do not monitor their goal progress and provides a conceptual structure for further studies of these reasons. Our findings suggest that the reasons that people most strongly endorse tend to be emotional; however, these reasons do not necessarily discourage progress monitoring. Rather, the present findings suggest that people seem to avoid monitoring their goal progress when they feel that information on their progress will not be accurate or useful, and when they feel more in control of their goal attainment.

## Author contributions

BC led the research, identified the research questions, designed the studies, analyzed the data, interpreted the findings, and prepared the manuscript for publication. TW was PI on the grant that funded this research and helped to identify the research questions, design the studies, interpret the findings, and prepare the manuscript for publication. YB helped to identify the research questions, design the studies, interpret the findings, and prepare the manuscript for publication.

## Funding

This research was funded by a grant from the European Research Council (ERC-2011-StG-280515).

### Conflict of interest statement

The authors declare that the research was conducted in the absence of any commercial or financial relationships that could be construed as a potential conflict of interest.

## Footnotes

^1^http://www.bps.org.uk/publications/policy-and-guidelines/research-guidelines-policy-documents/research-guidelines-poli.

^2^This method was chosen rather than computing factor scores based on the regression method so that we could compare the extent to which participants endorsed each of the reasons for not monitoring reflected by the different factors.

^3^In fact, the progress calculator was fictional and so, once participants who choose to access the calculator had provided their information, they were informed that it was not actually possible to calculate their progress. However, participants were provided with a link to a website that would allow them to calculate the number of calories that they would need to consume daily in order to achieve their weight loss goal. All participants were also fully debriefed as to the true purpose of the research at the end of the study.

^4^We thank the reviewer for this suggestion.
